# Effects of folic acid on body folate status, ovarian function, and organ indexes of offspring during embryonic stage

**DOI:** 10.3389/fvets.2025.1564592

**Published:** 2025-06-10

**Authors:** Jianfei Zhao, Zhongqian Lu, Jialin Wu, Yufei Zhu

**Affiliations:** ^1^College of Life Sciences and Agri-forestry, Southwest University of Science Technology, Mianyang, China; ^2^Shanxi Dayu Bioengineering Co., Ltd., Yuncheng, China; ^3^DAYU Bioengineering (Xi’an) Industrial Development Research Institute, Xi’an, China

**Keywords:** folic acid, female broiler breeder, folic acid transporter, offspring, ovarian function

## Abstract

This experiment aimed to investigate the effect of dietary folic acid supplementation on maternal folate status and ovarian function in female broiler breeders. Fifty 30-week-old Arbor Acres female broiler breeders were randomly divided into two groups (5 pens/group, 5 hens/pen) after 2 weeks of pre-feeding: the control group (Con, supplementation 0 mg/kg folic acid) and the folic acid group (FA, supplementation 4 mg/kg folic acid). The trial lasted 16 weeks. Folate content in eggs, maternal plasma, and offspring plasma (1-day-old) was measured, alongside expression levels of folate transporters in the ovary, liver, duodenum, and jejunum. Ovarian transcriptome analysis was performed. Results showed that the FA group had significantly increased folate deposition in eggs (*p* < 0.05) and offspring plasma folate levels (*p* < 0.01). No significant differences were observed in embryonic organ indexes (*p* > 0.05). The FA group had downregulated mRNA expression of proton-coupled folate transporter (*PCFT*) in the jejunum, folate transporter (*RFC*) in the liver, and folate receptors (*FR*) in the ovary (*p* < 0.05). However, they had upregulated *RFC* in the duodenum (*p* < 0.05). Transcriptome analysis identified 326 differentially expressed genes (217 up-regulated, 109 down-regulated; *p* < 0.05). KEGG enrichment revealed 10 pathways, including cell cycle, Wnt signaling, and steroid biosynthesis. These findings suggest that folic acid enhances ovarian reproductive gene expression and improves folate transfer to eggs and offspring.

## 1 Introduction

Feed restriction has been necessary for broiler breeders during the rearing and laying periods to prolong breeding duration, improve egg quality, and maintain metabolic disturbances (1, 2). As oviparous species, poultry embryos rely entirely on nutrients deposited during egg formation, making maternal nutrition a critical determinant of offspring embryonic development and post-hatch growth (3). Vitamins play indispensable roles in poultry development, health, and reproductive efficiency (4). Among these, folic acid (vitamin B9), a synthetic form of folate, is essential for DNA synthesis, methylation, and cellular proliferation. Unlike plants and microorganisms, humans, animals, and birds lack the capacity for *de novo* folate synthesis, necessitating lifelong exogenous intake. Beyond folic acid systemic benefits, folate metabolism critically regulates ovarian function, embryogenesis, and pregnancy maintenance (5). For instance, embryonic folic acid administration alters epigenetic modifications, thereby promoting developmental trajectories (6) and improving post-hatch immune competence and productivity (7). These findings underscore the potential of early folic acid intervention to modulate offspring phenotypes. However, current research on folic acid in poultry nutrition predominantly focuses on commercial broilers during growth phases, with limited exploration of its role in maternal-fetal folate transfer or ovarian transcriptional regulation in breeders. This study aimed to bridge this gap by investigating how dietary folic acid supplementation influences maternal folate status, ovarian transcriptome dynamics, and offspring developmental outcomes, thereby providing insights into optimizing the nutrition of female broiler breeders.

## 2 Materials and methods

The animal experimental procedures were approved by the Institutional Animal Care and Use Committee of Southwest University of Science and Technology (Permit Number: L2023013).

### 2.1 Experimental design

**Figure 1:**
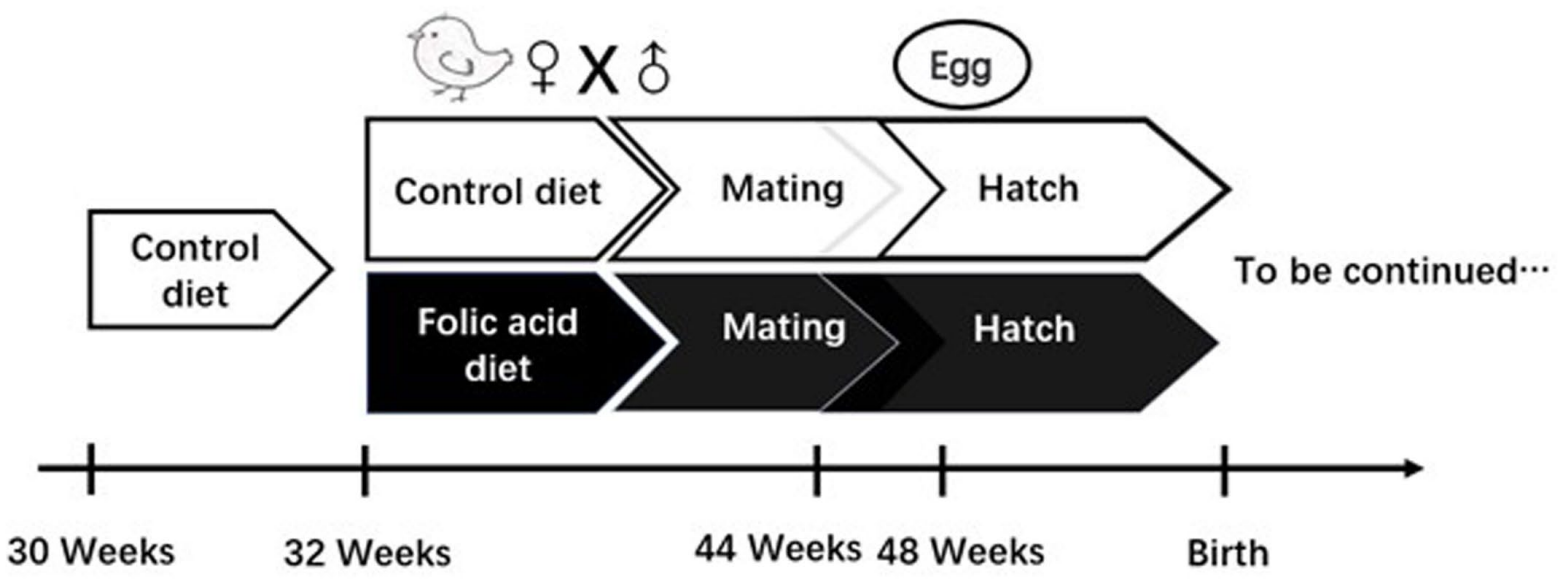
Study design investigating effects of folic acid on female broiler breeders.

The experimental design of this study is shown in Figure 1, and detailed descriptions are as follows: a total of 50 Arbor Acres female broiler breeders were purchased from Shaanxi Huaqin Broiler Chicken Co. Ltd. (Lantian, China). After 2 weeks of pre-feeding, female broiler breeders were stratified by body weight and randomly assigned to two groups using a random number generator (Microsoft Excel). Each group contained 5 pens (5 female broiler breeders/pen), with pens distributed across the housing facility to minimize environmental bias. Each replicate was randomly assigned to receive one of two dietary folic acid levels: (1) a control diet with no supplemental folic acid or (2) a control diet + 4 mg folic acid/kg of diet. The treatment time was 16 weeks. The basal diet was a corn-soybean meal ration formulated to feed the requirements of female broiler breeders consuming 160 g/day. Experimental diets were referenced to the nutritional requirements of NRC (1994); the composition and nutritional levels are shown in Table 1. The light duration was 16 h, and artificial insemination was performed every 3 days at a ratio of 1 male to 10 females. The 7-day-old eggs were collected at 44 weeks of age for incubation, and the eggs were stored in a constant temperature refrigerator at 16–18°C and a relative humidity of 60–65% before hatching. The eggs were incubated using an automatic 96 incubator (9TV-2A, Beijing Blue Sky Electronic Technology Co., Ltd.). The incubation procedure is shown in Table 2.

**Table 1:** Composition and nutrient levels of the diet for female broiler breeders (air-dry basis).

Items	Control group	FA group
Ingredients (%)		
Corn	60.00	60.00
Soybean meal	21.00	21.00
Wheat bran	6.93	6.93
Soybean oil	2.20	2.20
Limestone	7.70	7.70
Dicalcium phosphate	1.20	1.20
Salt	0.30	0.30
*L*-Lys·HCl	0.12	0.12
*DL*-Met	0.13	0.13
Thr	0.07	0.07
Mineral premix^a^	0.15	0.15
Vitamin, including	0.20	0.20
Vitamin A (IU/kg)	12000.00	12000.00
Vitamin D 3 (IU/kg)	3500.00	3500.00
Vitamin E (IU/kg)	100.00	100.00
Vitamin B 1 (mg/kg)	6.60	6.60
Vitamin B 2 (mg/kg)	12.00	12.00
Vitamin K (mg/kg)	4.40	4.40
Niacin (mg/kg)	50.00	50.00
Pantothenic acid (mg/kg)	15.50	15.50
Vitamin B 6 (mg/kg)	4.40	4.40
Biotin (μg/kg)	220.00	220.00
Folic acid (mg/kg)	0.00	4.00
Vitamin B 12 (μg/kg)	22.00	22.00
Choline (mg/kg)	1210.00	1210.00
Nutrition composition^b^		
Metabolizable energy (MJ/kg)	11.34	11.34
Crude protein	15.45	15.45
Lys	0.75	0.75
Met + Cys	0.62	0.62
Thr	0.55	0.55
Calcium	3.20	3.20
Total phosphorus	0.63	0.63
Available P	0.36	0.36

a Mineral premix provided the following per kg of diets: Mn 100 mg, Zn 80 mg, Fe80 mg, Cu 8 mg, I 1 mg, Se 0.30 mg.

b Nutrient levels were calculated values.

**Table 2:** Incubation procedures.

Incubation phase	Temperature (°C)	Humidity (%)	Egg turning
E1–E6	38.0	60	Turn the eggs every 1.5 h, for 180 s each time
E7–E12	37.8	55	
E13–E18	37.6	60	
E19–E21	37.2	70	Stop turning

### 2.2 Samples collection

At the 48th week, one female broiler breeder per replicate was randomly selected using a randomization protocol. Blood samples were collected from the wing vein, and birds were humanely euthanized following approved animal welfare guidelines. Ovary, liver, duodenum, and jejunum were promptly dissected, and residual blood or intestinal contents were thoroughly rinsed with pre-chilled phosphate-buffered saline (PBS, 4°C). Tissues were flash-frozen in liquid nitrogen to prevent RNA degradation and stored at −80°C until mRNA extraction and analysis.

### 2.3 Chemical analysis

The content of 5-methyltetrahydrofolate (5-MTHF) in egg yolk was determined according to the method of House et al. (8). The yolks of egg samples were separated, freeze-dried, and stored at −20°C until analysis. Quantitation of 5-MTHF content of egg yolk samples after extraction into an ascorbate buffer (pH 7.4) was analyzed for 5-MTHF via reverse-phase HPLC with fluorescence detection using the method. The purified 5-MTHF folate external standard curve was used to quantify egg folate concentration. The amount of egg folate was expressed as micrograms of folate per egg.

The analytical standard of 5-MTHF (CAS Number: 26560-38-3, Purity: 98%) was purchased from Sigma (St. Louis, MO, United States). HPLC separation was performed with a Shimadzu LC-20 instrument equipped with HPLC-UV system. Folate was separated on an HC-C18 column (250 mm × 4.6 mm, 5 μm) (Agilent, United States), and its temperature was set at 27°C. The mobile phase was phosphate buffer at pH 2.3 (A) and acetonitrile (B) at a flow rate of 0.8 mL/min. The gradient was as follows: 0–4 min, 9% B, 4–12 min, 24% B, 12–14 min, 24% B, and 14–15 min, 9% B. The injection volume was 20 μL, and the autosampler temperature was 27°C. The contents of 5-MTHF and homocysteine (Hcy) in plasma were detected using Elisa (ED-60659, ED-60216, Lun Chang Shuo Biotech, Xiamen, China).

### 2.4 Embryonic weight and organ indexes

After the embryo or chick was slaughtered, the weight of the embryo or chick, residual yolk, and liver was measured. The remaining yolk weight was expressed relative to the initial egg weight. The organ index was described as the relative weight of the organ weight to the embryo weight without yolk.

### 2.5 qRT-PCR and RNA-seq analysis

Total RNA of the ovary, liver, and intestinal mucosa (duodenum and jejunum) was extracted using the Trizol Reagent protocol (AG21102, AG, Changsha, China). The concentration, purity, and integrity of RNA samples were verified, and cDNA was synthesized using the *Evo M-MLV* RT Kit for qPCR (AG11707, AG, Changsha, China). The mRNA expression of reduced folate carrier (*RFC*), folate receptor (*FR*) in the ovary, and the mRNA expression of proton-coupled folate transporter (*PCFT*) and RFC in duodenum and jejunum were analyzed with a SYBR^®^ Green Premix Pro Taq HS qPCR Kit (AG11701, AG, Changsha, China) on the iCycler IQ5 (Bio-Rad, Hercules, CA, United States). The primers are listed in Table 2. The total volume of the reaction system was 10 μL: 5 μL of SYBR Green Premix Pro Taq, 0.5 μL upstream primers (10 pmol/μL), 0.5 μL downstream primers (10 pmol/μL), and 4 mL cDNA. The reactions of real-time PCR were carried out at 95°C for 30 s, followed by 40 cycles at 95°C for 5 s, and at 60°C for 30 s. All samples were run in triplicate, and the average cycle threshold (Ct) values were normalized to *β-actin* and quantified by the 2^−ΔΔCt^ method (9). Finally, 2^−ΔΔCt^ values were normalized to the control group. In total, 10 RNA-seq libraries (5 control groups and 5 folic acid groups) were constructed. Clean reads were obtained by removing contained adaptor contamination, low-quality, and undetermined bases. Then, the clean reads sequence quality was assessed by Q20, Q30, and GC content. All downstream analyses were based on clean high-quality data. The difference expression analysis between the two comparison combinations was conducted using DESeq software (1.20.0). The conditions for screening differentially expressed genes were expression difference multiple |log2FoldChange| >2, significance *p* < 0.05. Personalbio Co., Ltd. commissioned the detection and analysis.

### 2.6 qRT-PCR verification

To ensure the reproducibility of differentially expressed genes obtained from RNA-seq data, six differential expressed genes (DEGs) were randomly selected for qRT-PCR verification in this experiment. The RNA samples used for qRT-PCR were the same as for RNA-seq. The primers are listed in Table 3.

**Table 3:** Primer sequence of target genes.

Gene	Accession number	Primer sequences (5′–3′)	Product size (bp)
*β-actin*	NM_205518.1	F: ATTGTCCACCGCAAATGCTTC	113
		R: AAATAAAGCCATGCCAATCTCGTC	
*PCFT*	NM_001205066.1	F: GGCTGTGCTCACTTGTGGCTA	153
		F: TGAAGATCCGTTGGGCACTG	
*RFC*	NM_001006513.1	R: GCTATCTGGAAAATAGCGATGGG	175
		R: GAAGGTTGGAGTCCTGGATTTCTAT	
*FR*	XM_015280910	F: CATCCAGGATATGTGCTTGTATGA	180
		R: CAGCCCTTGTGCCAGTTCTC	
*MYL3*	NM_205159.2	F: ACCTAAGAAGGCGCCTGAAC	153
		R: TGAGAACGCTTCCTTAAATTCTTCA	
*IHH*	NM_204957.3	F: ACAGGGACCGCAACAAGT	120
		R: CAGCCGAGTGCTCTGACT	
*WNT4*	NM_204783.1	F: TGTGACCACGACCTCAAGAA	160
		R: ACCAGTGGAATTTGCAGCTG	
*IL7*	NM_001398241.1	F: AGCCTTCGATCTTGCTGGAAT	161
		R: ATCCAAATCCGGCACAAGGT	
*GDF6*	XM_015282935	F: ACCGGACGGTACTCCAACTA	102
		R: CAGAGCTGCTGAACCGAAGA	
*MYH11*	NM_205274.3	F: AGCACAACTGCCTTGTCTCC	196
		R: CCTGCAACAGTCCTACAAATCC	

*PCFT*, proton-coupled folate transporter; *RFC*, reduced folate carrier; *FR*, folate receptor; *MYL3*, myosin light chain 3; *IHH*, Indian hedgehog; *WNT4*, WNT family member 4; *IL7*, interleukin-7; *GDF6*, growth differentiation factor 6; *MYH11*, myosin heavy chain 11.

### 2.7 Statistical analysis

A t-test examined the significant differences between the control and the folic acid groups. All data were analyzed using SPSS 21.0 and expressed as mean ± SEM. Significance levels: ^*^p < 0.05 and ^**^p < 0.01.

## 3 Results

### 3.1 5-MTHF content of yolk and plasma

**Figure 2:**
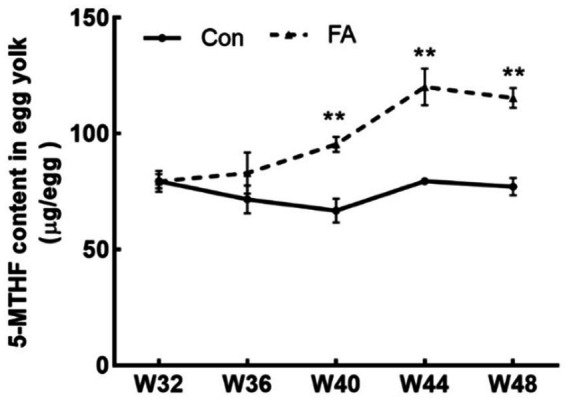
Effects of dietary folic acid on 5-MTHF content in eggs of female broiler breeders (*n* = 5). Values are means ± SEM, ^**^p < 0.01. Con, control group, FA, folic acid.

**Figure 3:**
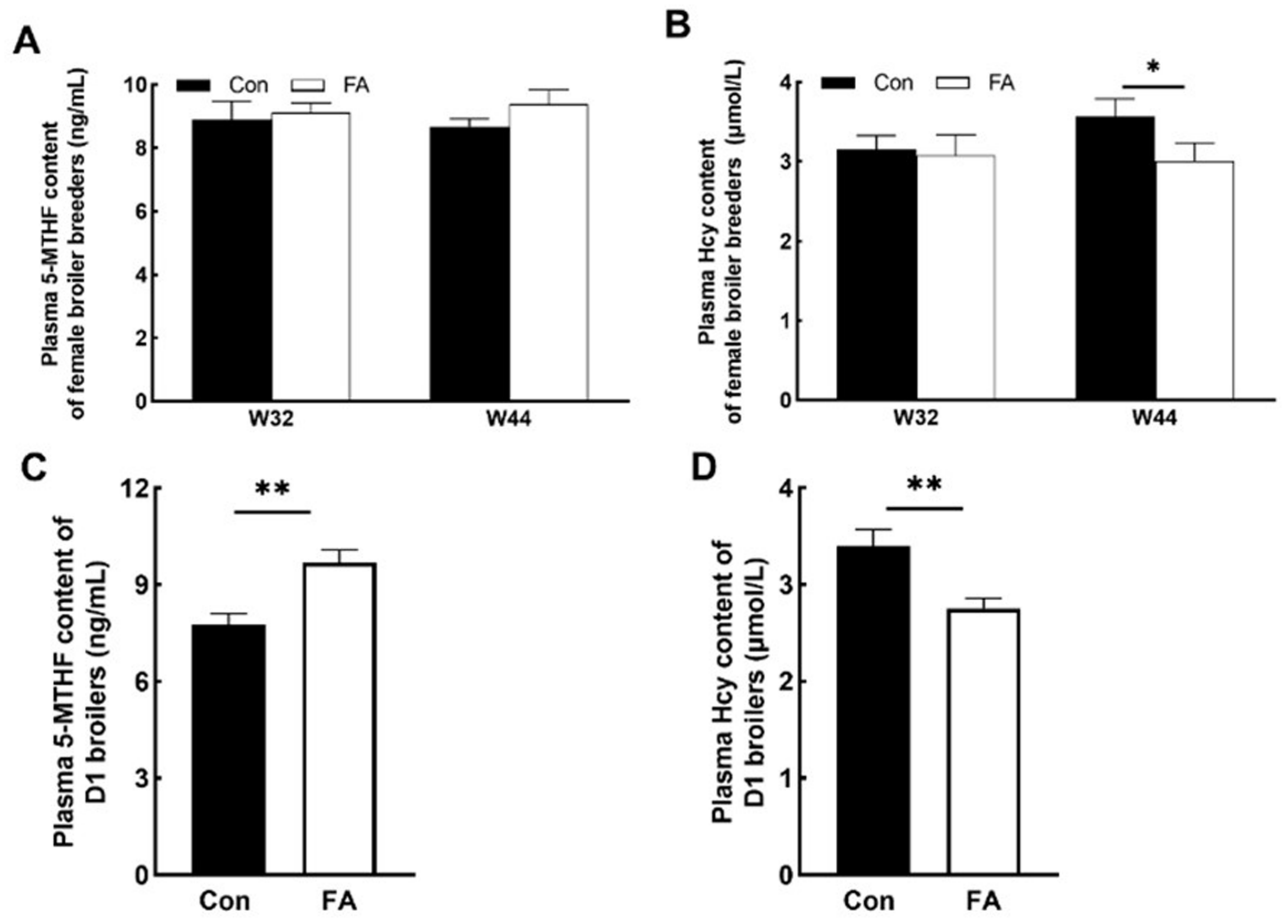
Effects of folic acid on 5-MTHF and Hcy content of female broiler breeders and offspring (*n* = 5). Values are means ± SEM, ^*^p < 0.05 and ^**^p < 0.01. Con, control group, FA, folic acid. **(A)** Plasma 5-MTHF content of female broiler breeders. **(B)** Plasma Hcy content of female broiler breeders. **(C)** Plasma 5-MTHF content of D1 broilers. **(D)** Plasma Hcy content of D1 broilers.

As shown in Figure 2, dietary folic acid supplementation significantly increased folate deposition in eggs (*p* < 0.01). As shown in Figure 3, for 32 weeks of age, there were no significant differences in plasma 5-MTHF and Hcy contents between the control group and the FA group. At 44 weeks of age, the plasma Hcy concentration of female broiler breeders in the folic acid supplementation group was significantly lower than that in the control group (*p* < 0.05); 5-MTHF content had no significant difference (*p* > 0.05) but was higher than the control group.

### 3.2 Embryonic development characteristics

As shown in Table 4, there was no significant difference in the embryo weight, liver index, or residual yolk index of the offspring on embryonic day 15 (E15), embryonic day 19 (E19), or postnatal 1st day (D1) when folic acid was added to the diet of female broiler breeders (*p* > 0.05).

**Table 4:** Effects of folic acid levels of female broiler breeders on offspring embryonic organ index.

Items	Age	Con	FA	*p*-value
Egg weight (g)	—	70.21 ± 0.91	69.29 ± 0.86	0.625
Relative weight of the embryo or chick (g)	E15	14.02 ± 0.68	14.30 ± 0.44	0.738
	E19	34.48 ± 0.66	32.45 ± 1.01	0.119
	D1	42.51 ± 0.96	40.20 ± 0.74	0.199
Liver index (%)	E15	1.57 ± 0.16	1.73 ± 0.06	0.384
	E19	1.79 ± 0.07	1.90 ± 0.06	0.281
	D1	3.05 ± 0.07	3.17 ± 0.08	0.276
Relative weight of the residual yolk (%)	E15	29.17 ± 2.28	25.85 ± 3.40	0.432
	E19	21.43 ± 0.64	20.88 ± 0.95	0.648
	D1	7.43 ± 0.48	8.27 ± 0.52	0.247

### 3.3 Regulation of folic acid on expression of folic acid transporters

**Figure 4:**
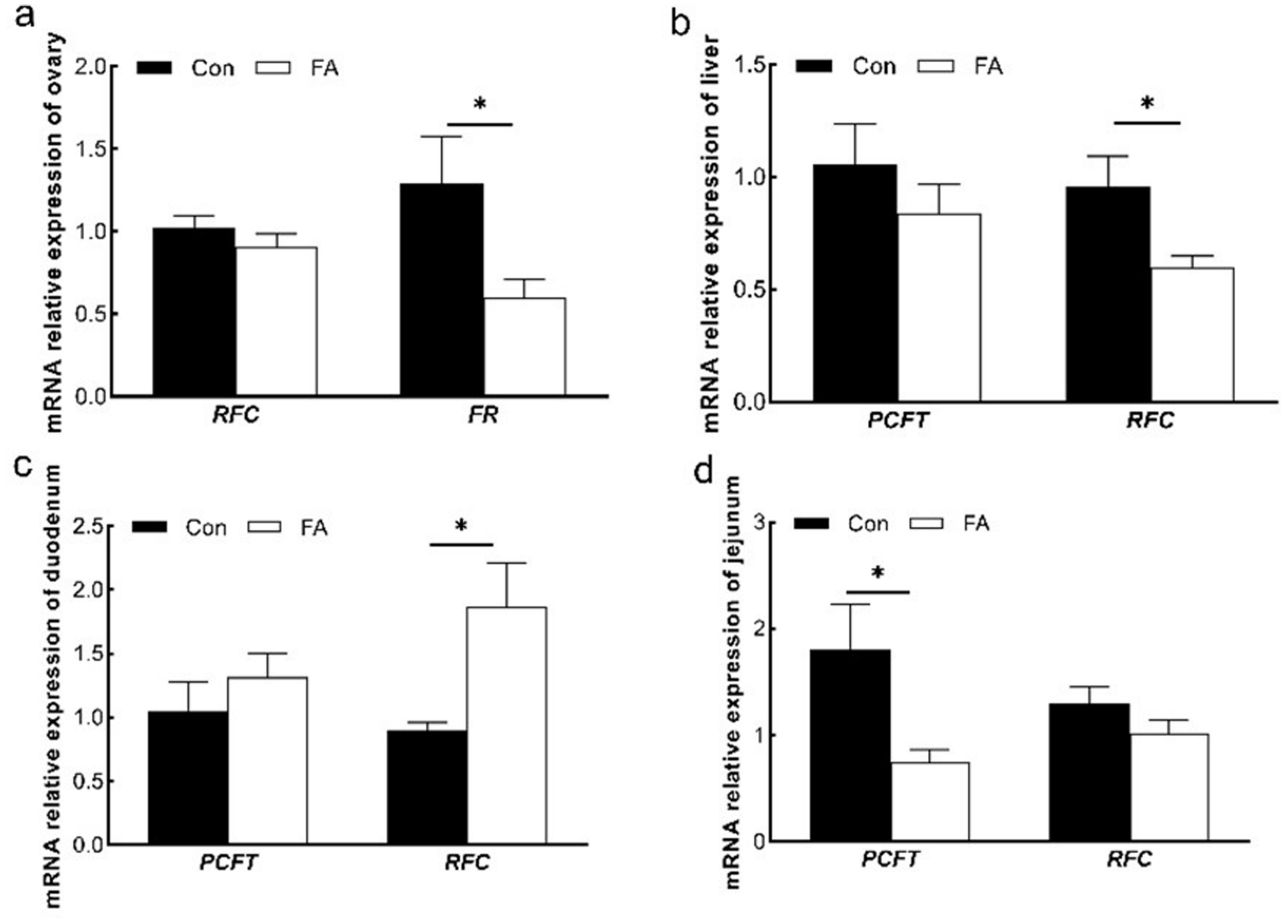
Effects of dietary folic acid on expression of folic acid transporter in ovary **(a)**, liver **(b)**, duodenum **(c)**, and jejunum **(d)** of female broiler breeders (*n* = 5). Values are means ± SEM, ^*^p < 0.05. Con, control group, FA, folic acid group; *FR*, folate receptor; *PCFT*, proton coupled folate transporter; *RFC*, reduced folate carrier.

Folic acid absorption in the foregut is mainly dependent on *PCFT* and *RFC*. As shown in Figure 4, the mRNA relative expression of *RFC* in the liver and *PCFT* in the jejunum was significantly decreased in folic acid (*p* < 0.05). In the duodenum, the relative expression of mRNA of *RFC* was increased considerably. *FR* and *RFC* play major roles in the ovary; the mRNA relative expression of *FR* was significantly decreased (*p* < 0.05).

### 3.4 Effects on the ovary transcriptome profile of female broiler breeders

In the study, we considered and determined the transcriptome profile of the ovary to investigate the effect of folic acid on ovary function because the folic acid supplementation was not significant for female broiler breeder’s performance.

**Figure 5:**
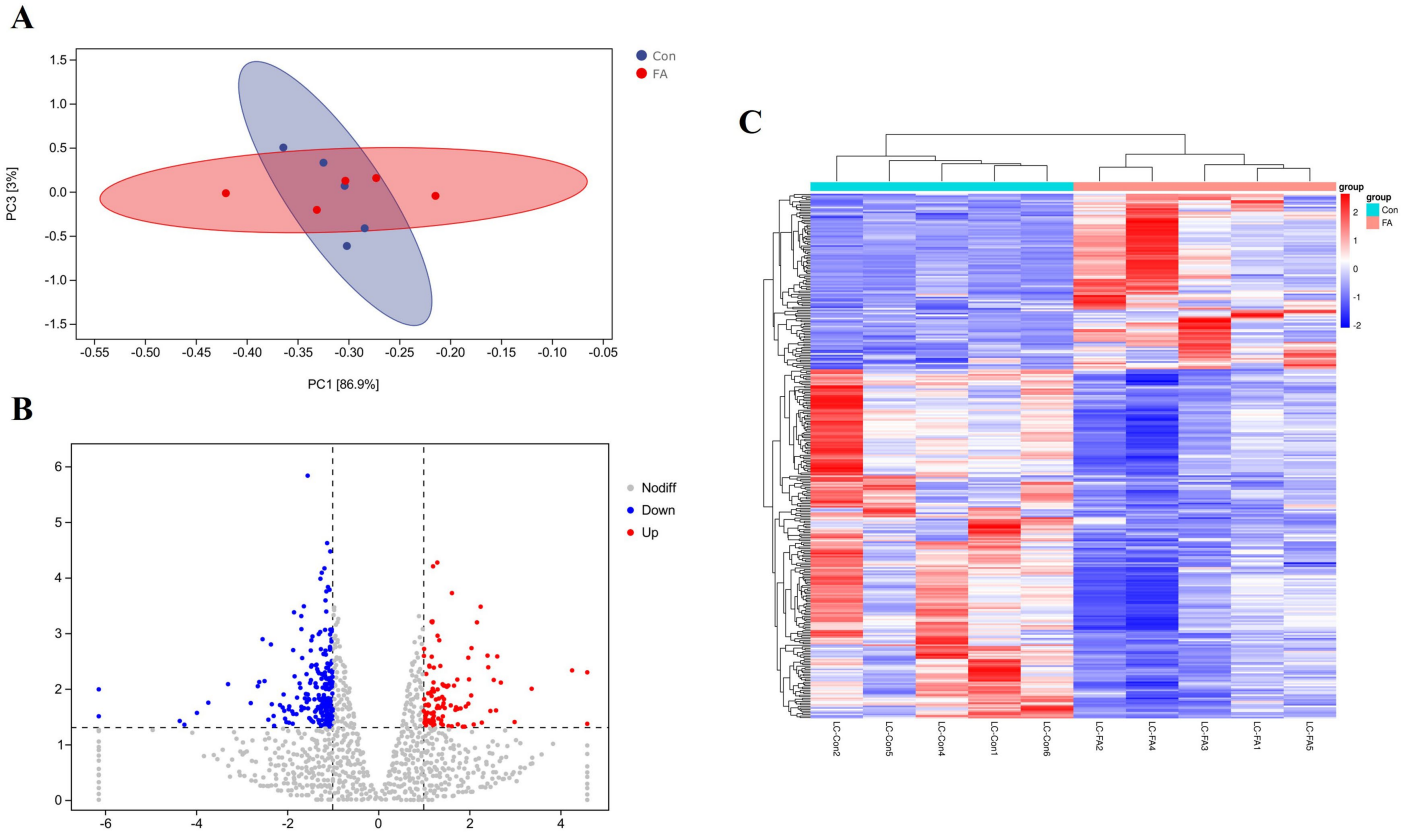
Differential expression analysis of folic acid on ovary transcriptome of female broiler breeders (*n* = 5). **(A)** Principal component analysis plot. **(B)** Volcano plot of differentially expressed mRNAs. **(C)** Clustering heat map of differentially expressed mRNAs. Red indicates highly expressed genes and green indicates lowly expressed genes.

This test uses *p* < 0.05 and a difference multiple of more than 2 times as the screening criteria, as shown in Figure 5. (A) The PCA model can effectively separate the differences between the folic acid addition and control groups in this experiment. (B) A total of 326 differentially expressed genes were screened out in the ovaries of female broiler breeders. Compared with the control group, there were 109 up-regulated differential genes and 217 down-regulated genes in the ovaries of the folic acid-supplemented group. (C) Heat map analysis characterizes the differential genes between the two groups, indicating good reproducibility between the repeated samples.

**Figure 6:**
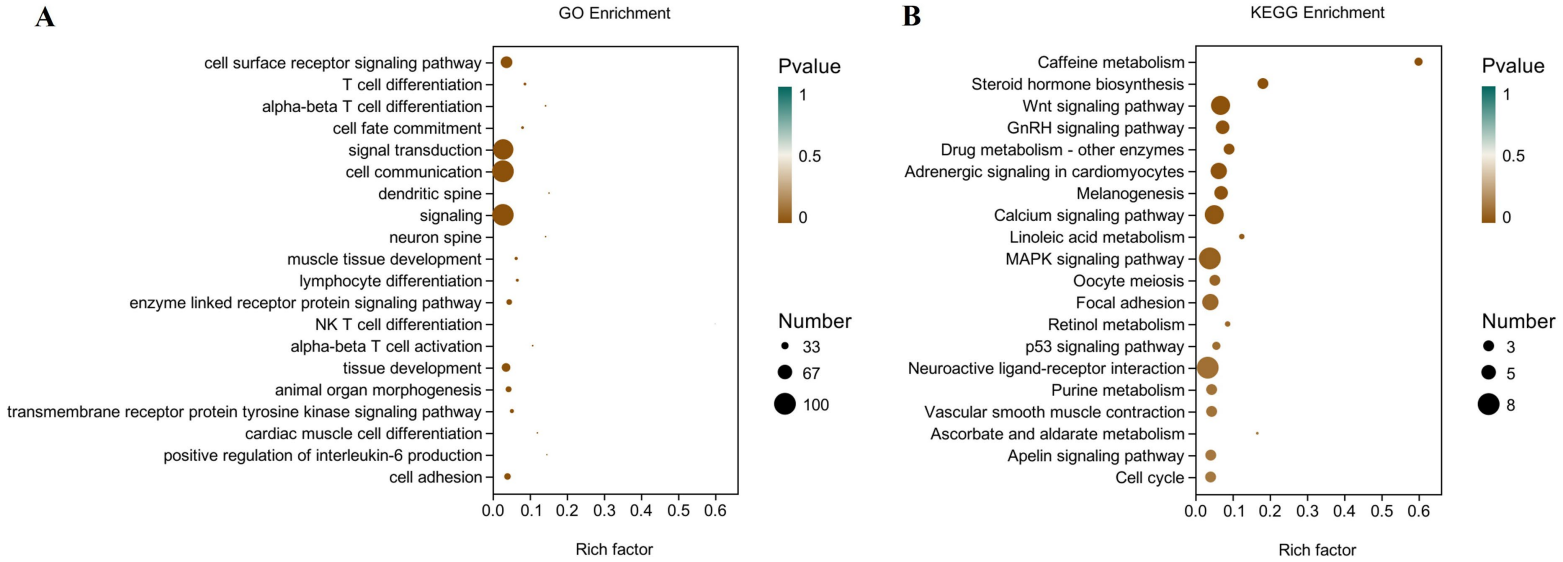
Scatter plot of GO and KEGG enrichment analysis of differentially expressed genes in the ovary of female broiler breeders (*n* = 5). **(A)** GO enrichment. **(B)** KEGG enrichment.

To define the functions of differentially expressed genes (DEGs) caused by folic acid, GO and KEGG were combined to examine the pathways involved (Figure 6). The most enriched GO terms were biological processes related to cell differentiation, signaling, and tissue development. Results of KEGG pathway analysis revealed that 10 signaling pathways were significantly enriched. The 10 considerably enriched signaling pathways involved in the regulation of folic acid are caffeine metabolism, steroid hormone biosynthesis, Wnt signaling pathway, gonadotropin signaling (GnRH) pathway, drug metabolism, adrenergic signaling in cardiomyocyte, melanogenesis, calcium signaling pathway, linoleic acid metabolism, and MAPK signaling pathway. Among them, Wnt signaling pathway, steroid biosynthesis signaling pathway, and GnRH pathway production may be involved in the reproductive process of female broiler breeders.

### 3.5 Validation of transcriptome by qRT-PCR

**Figure 7:**
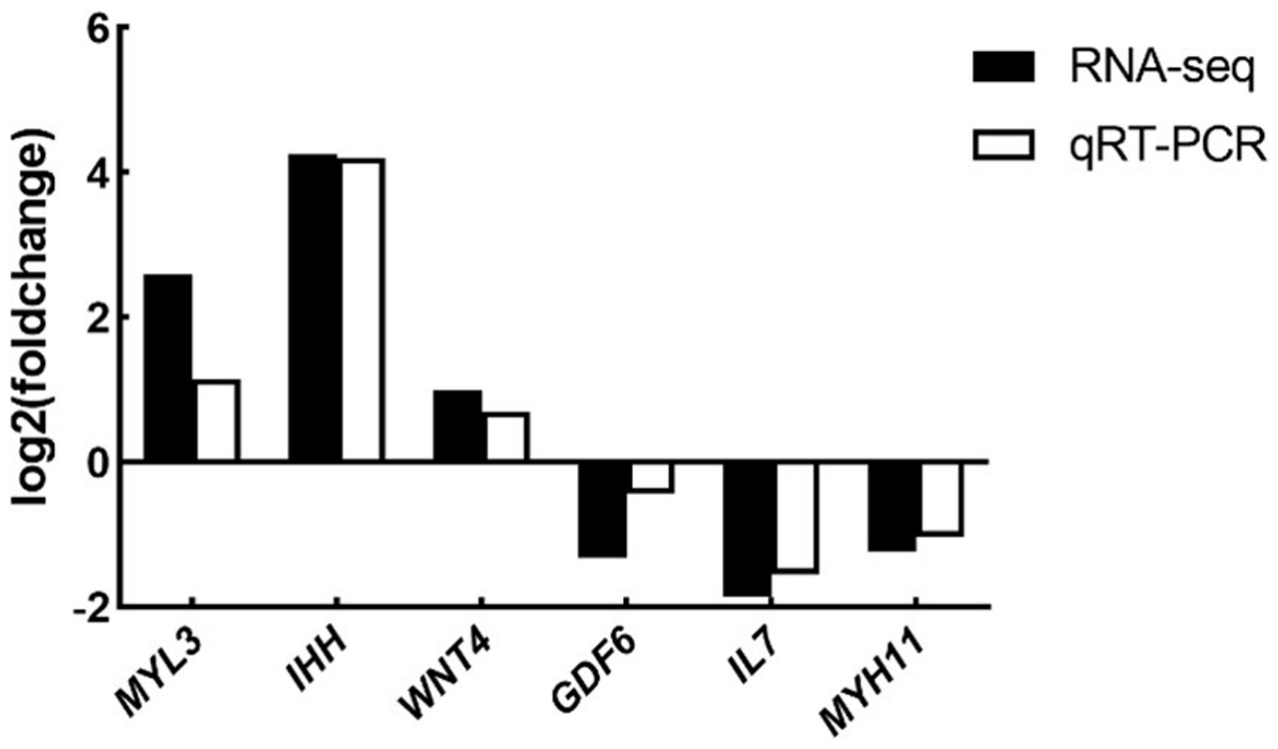
Expression levels of selected DEGs quantified by qRT-PCR (*n* = 5). Differential expression analysis of folic acid on ovary transcriptome of female broiler breeders (*n* = 5). *MYL3*, myosin light chain 3; *IHH*, Indian hedgehog; *WNT4*, WNT family member 4; *IL7*, interleukin-7; *GDF6*: growth differentiation factor 6; *MYH11*, myosin heavy chain 11.

To validate the RNA-seq data, the expression levels of six DEGs (namely, *MYL3*, *IHH*, *WNT4*, *IL7*, *GDF6*, and *MYH11*) were determined by qRT-PCR. Although the expression amplitudes of RNA-seq and qRT-PCR are different, the expression regulation trends in the two technologies are consistent (Figure 7). Correlation analysis showed that the expression levels of the validated genes were highly correlated between RNA-seq and qRT-PCR. Therefore, the results of RNA-seq are reliable and consistent with the results of qRT-PCR analysis.

## 4 Discussion

This study demonstrates that dietary folic acid supplementation significantly increases folate deposition in hatching eggs, consistent with prior findings (10–12). Specifically, yolk 5-methyltetrahydrofolate (5-MTHF) increased by 120% in the FA group compared to controls (Con), reaching 120 μg/egg. While earlier studies reported lower deposition levels (54.5 μg/egg with 15 mg/kg supplementation) (13), this discrepancy likely stems from differences in detection methodologies (HPLC vs. ELISA) and egg weight variations. Notably, folate deposition exhibited saturation kinetics, as higher dietary folate levels did not proportionally increase egg folate content, suggesting a transport-limited mechanism. Blood folate serves as the precursor pool for egg deposition, and the elevated plasma-to-egg folate ratio observed here confirms efficient maternal-fetal transfer (8, 10). High levels of Hcy can easily lead to follicular atresia, reduce the quantity and quality of oocytes, and affect early embryonic development (14). In this experiment, folate metabolism reduced plasma Hcy levels while elevating methionine availability.

Intestinal folate absorption is dynamically regulated by substrate concentration. In this study, folic acid supplementation downregulated mRNA expression of *RFC* and *PCFT* in the jejunum and liver, contrasting with duodenal *RFC* upregulation. This aligns with reports showing dose-dependent duodenal *RFC* responses (15, 16) and jejunal suppression under high folate (17, 18). We propose that folate concentration dictates transporter prioritization, with *RFC* dominating at moderate levels and passive diffusion prevailing at saturation. In ovarian tissue, folic acid significantly reduced *FR* expression, potentially reflecting epigenetic silencing via folate-driven DNA hypermethylation (19). Paradoxically, despite *FR* downregulation, ovarian folate deposition increased, implying alternative transport mechanisms. Elevated *FR* in ovarian cancer (20) and its suppression here suggest folate sufficiency may mitigate pathological overactivation, preserving follicular integrity.

The ovary orchestrates egg production through folliculogenesis and hormonal regulation. Egg weight significantly influences both hatching rates and chick viability (21). RNA-seq analysis identified 326 DEGs, with significant enrichment in five pathways. Among them, neuroactive ligand-receptor interaction may be the most important pathway leading to the difference in egg production rate between high- and low-producing laying hens. Six DEGs (*GRPR*, *GRP*, *P2RX2*, *GALR1L*, *ADORA1*, and *MTNR1A*) were implicated in modulating neurotransmitter signaling, potentially enhancing hypothalamic-pituitary-ovarian (HPO) axis activity and ovulation frequency (22, 23). The steroid biosynthetic pathway, a known target of endocrine-disrupting chemicals (24), was significantly enriched in high-yielding ducks (25, 26). Ovarian steroid hormones, such as estrogen, regulate follicular dynamics by balancing proliferation and apoptosis signals (27). Concurrently, gonadotropin-releasing hormone (GnRH) coordinates the HPO axis to regulate gonadotropin secretion and sex hormone biosynthesis (28–30). GnRH pathway activation may explain improved follicular maturation in the FA group female broiler breeders. Mechanistically, upregulation of *WNT4* and GnRH pathway genes suggests that folate promotes follicular maturation and steroidogenesis. *WNT4* activates β-catenin signaling, which synergizes with FSH to stimulate granulosa cell proliferation and estrogen synthesis (31). Paradoxically, folate supplementation suppressed *FR* expression, likely via DNA hypermethylation at CpG islands in the *FRα* promoter, a phenomenon linked to reduced ovarian cancer risk (19). Despite *FR* downregulation, ovarian folate deposition increased, suggesting compensatory mechanisms such as *RFC*-mediated transport or passive diffusion at high folate concentrations. Collectively, these mechanisms enhance follicular reserve and oocyte quality, aligning with increased plasma folate deposition in eggs. Our findings position folic acid as a multi-target regulator of ovarian function, enhancing reproductive efficiency through epigenetic, transcriptional, and metabolic pathways.

## 5 Conclusion

Folic acid can increase the expression of genes related to ovarian reproductive function and promote the deposition of folic acid in breeding eggs and offspring. In summary, folic acid has a positive regulatory effect on ovarian function in female broiler breeders.

## Data Availability

The data presented in the study are deposited in the NCBI SRA repository, accession number PRJNA1269787.
